# Morphine—Ether Narcosis

**Published:** 1897-04

**Authors:** 


### MORPINE-ETHER NARCOSIS.

Riedel (*Berl. Klin. Woch.*, September 28, 1896), alluding to the fact that there are more sequelæ (such as bronchitis, etc.) after ether than after chloroform narcosis, states that in the former, cardiac paralysis and sudden arrest of the respiration do not occur. To guard against the danger of bronchitis, the sensibility of the bronchial mucous membrane should be diminished in order to lessen secretion. For this purpose the author gives an injection of morphine before the administration of ether. He has come to use this method more and more widely. Before the fourteenth year, however, the morphine is omitted. From 13 to 18 years of age, 0.005 gramme ($\frac{1}{12}$ grain) is given, and to older persons, 0.01 gramme ($\frac{1}{6}$ grain). This amount may not be sufficient in drunkards. Half an hour after the injection, the ether is administered cautiously, only 3 or 4 grammes (45 to 60 grains) being poured on the inner side of Julliard's mask. The mask is not pressed on the face at once; so that time is allowed for the patient to get used to the ether, and thus there is no tendency to cough. The mask has a cover of oiled paper, and a few drops of ether are from time to time poured through the opening in it. If the operation only takes an hour the effects of the morphine will last, and very little ether is necessary, but in longer operations much more ether may be required. Sleep is prolonged after the operation for a considerable time. The author has never observed bronchitis in children after ether. He has employed this method of anæsthesia for operation on all parts of the body during the past three years. If it were not for bronchitis, ether would be an ideal anæsthetic. Even fifty years after the discovery of chloroform a safer anæsthetic than chloroform is wanted. At present he observes that we must be satisfied with ether, which he looks upon as the best available anæsthetic. In only one case was death directly attributable to the ether, and the patient was here the subject of emphysema. There have been isolated cases of pneumonia, mostly in elderly and cachectic persons, and not to be put down to the ether. Ether exercises an irritant action upon the bronchi, and Riedel believes that the greater part of its dangerous properties may be avoided by the above-described mode of administration.—*British Medical Journal*, November 14, 1896.

